# Establishment of a Virus-Induced Gene Silencing (VIGS) System in *Syringa villosa* and Functional Characterization of the Floral Scent-Associated Gene *SvTPS3*

**DOI:** 10.3390/plants15203179

**Published:** 2026-10-16

**Authors:** Yalan Liu, Yuguang Liu, Jia Cui, Ruxu Gao, Yajie Meng, Xiaohui Zhou, Mengran Jin, Baosheng Shi

**Affiliations:** 1 Hebei Key Laboratory of Floral Biological Breeding, College of Landscape and Tourism, Hebei Agricultural University, Baoding 071000, China; liuyalan1116@163.com (Y.L.); liuyuguang@hebau.edu.cn (Y.L.); 15227286191@163.com (J.C.); 19868823801@163.com (R.G.); mengyajie2000@163.com (Y.M.); zhouxh@hebau.edu.cn (X.Z.); 2 Department of Agricultural and Animal Husbandry Engineering, Cangzhou Technical College, Cangzhou 061001, China

**Keywords:** *Syringa villosa*, Virus-induced gene silencing, phytoene desaturase gene, floral fragrance, monoterpene synthase gene

## Abstract

Functional genomic studies in woody ornamental plants are often constrained by the lack of effective gene function validation tools. Virus-induced gene silencing (VIGS) provides a rapid reverse-genetic approach for functional analysis, particularly in plant species where stable transformation remains challenging. However, a tissue-applicable VIGS system has not yet been established for *Syringa villosa*, an important woody ornamental species with valuable floral traits. In this study, an *Agrobacterium*-mediated VIGS system was established and optimized in *S. villosa* using the phytoene desaturase gene (*SvPDS*) as a reporter gene. Different inoculation methods and *Agrobacterium* concentrations were evaluated to determine suitable conditions for gene silencing. Syringe infiltration with an *Agrobacterium* suspension at an OD_600_ of 0.8 achieved the highest *SvPDS* silencing efficiency (80.00%) and showed good applicability for subsequent functional studies. The optimized VIGS system was further extended from vegetative tissues to floral tissues and applied to functional analysis of the candidate terpene synthase gene *SvTPS3*. Silencing of *SvTPS3* significantly reduced transcript abundance and decreased the emission rate of (E)-β-ocimene, the predominant volatile compound in *S. villosa* flowers. This study establishes a VIGS platform applicable to both vegetative and floral tissues of *S. villosa* and provides a valuable tool for functional characterization of genes associated with floral traits and secondary metabolism in woody ornamental plants.

## 1. Introduction

*Syringa villosa* is an ornamental shrub belonging to the genus *Syringa* and exhibits strong ecological adaptability, making it a valuable resource for studying the molecular basis of ornamental traits [1,2]. It is characterized by bright flowers, abundant blooming, and a pleasant fragrance, which contribute to its high ornamental value. However, as a woody plant, *S. villosa* remains difficult to genetically transform due to limitations in regeneration efficiency and transformation procedures. The lack of an efficient genetic transformation system restricts functional characterization of genes involved in floral traits, secondary metabolism, and other biological processes.

Floral scent is an important ornamental trait and is determined by a complex mixture of volatile organic compounds, among which terpenoids represent one of the most abundant and diverse groups of floral volatiles [3,4]. Plant terpenoids, including monoterpenes and sesquiterpenes such as β-ocimene, linalool, caryophyllene, and nerolidol, contribute not only to species-specific floral fragrance but also to ecological interactions between plants and their associated organisms [5,6,7]. The biosynthesis of volatile terpenoids is largely regulated by terpene synthases (TPSs), which catalyze the formation of diverse terpene skeletons from prenyl diphosphate precursors. Among floral volatile terpenoids, (E)-β-ocimene is widely distributed in ornamental plants and represents an important component of floral scent profiles. Previous studies have demonstrated that TPS genes contribute to β-ocimene formation in several species. For example, OfTPS3 and OfTPS1 from *Osmanthus fragrans* have been associated with the production of (E)-β-ocimene and β-linalool, respectively [8]. In addition, ocimene is one of the major volatile terpenes in *Syringa* oblata, and several TPS genes have been suggested to participate in its formation [9,10,11,12]. These findings indicate that TPS genes play important roles in determining floral terpene composition. However, functional characterization of TPS genes involved in floral scent formation remains limited in *S. villosa* due to the lack of efficient genetic manipulation tools.

Virus-induced gene silencing (VIGS) is an efficient reverse-genetic approach that enables transient suppression of target gene expression without requiring stable genetic transformation. In VIGS systems, a fragment of the target gene is inserted into a viral vector, and infection with the recombinant virus activates the plant RNA silencing machinery, resulting in sequence-specific degradation or suppression of target transcripts [13,14,15,16]. Compared with stable transformation, gene knockout, and antisense approaches, VIGS offers advantages including rapid implementation, simple operation, and applicability for functional analysis of individual genes or gene families [17,18,19,20]. VIGS systems have been established in diverse plant species, including Solanaceae, Poaceae, Brassicaceae, Malvaceae, and Rosaceae plants, and have been widely applied in studies of development, stress responses, pigmentation, and secondary metabolism [21,22,23,24,25,26,27]. In ornamental plants, VIGS has been successfully used to investigate genes involved in flower color formation, secondary metabolism, and other important traits [28,29,30,31,32,33]. Although VIGS approaches have been applied in several *Syringa* species, a systematic and optimized VIGS system for *Syringa villosa* has not yet been established. Therefore, developing an effective VIGS platform for *S. villosa* would provide an important tool for functional characterization of genes associated with floral traits and secondary metabolism. Phytoene desaturase (PDS), a key enzyme involved in carotenoid biosynthesis, is widely used as a reporter gene in VIGS studies because its silencing produces a visible photobleaching phenotype, allowing rapid evaluation of viral infection and silencing efficiency.

Therefore, establishing an efficient and reliable VIGS system for *S. villosa* is essential for functional genomic studies of this woody ornamental species. The aim of this study was to establish and optimize an *Agrobacterium*-mediated VIGS system in *S. villosa* using *SvPDS* as a reporter gene, and to evaluate the effects of different inoculation methods and *Agrobacterium* concentrations on VIGS efficiency. Furthermore, the applicability of the optimized VIGS system was assessed by targeting the candidate terpene synthase gene *SvTPS3* in floral tissues, providing a platform for future functional characterization of genes associated with floral scent biosynthesis and secondary metabolism in *S. villosa*.

## 2. Results

### 2.1. Verification of VIGS Vectors for SvPDS and SvTPS3 in S. villosa

**Figure 1:**
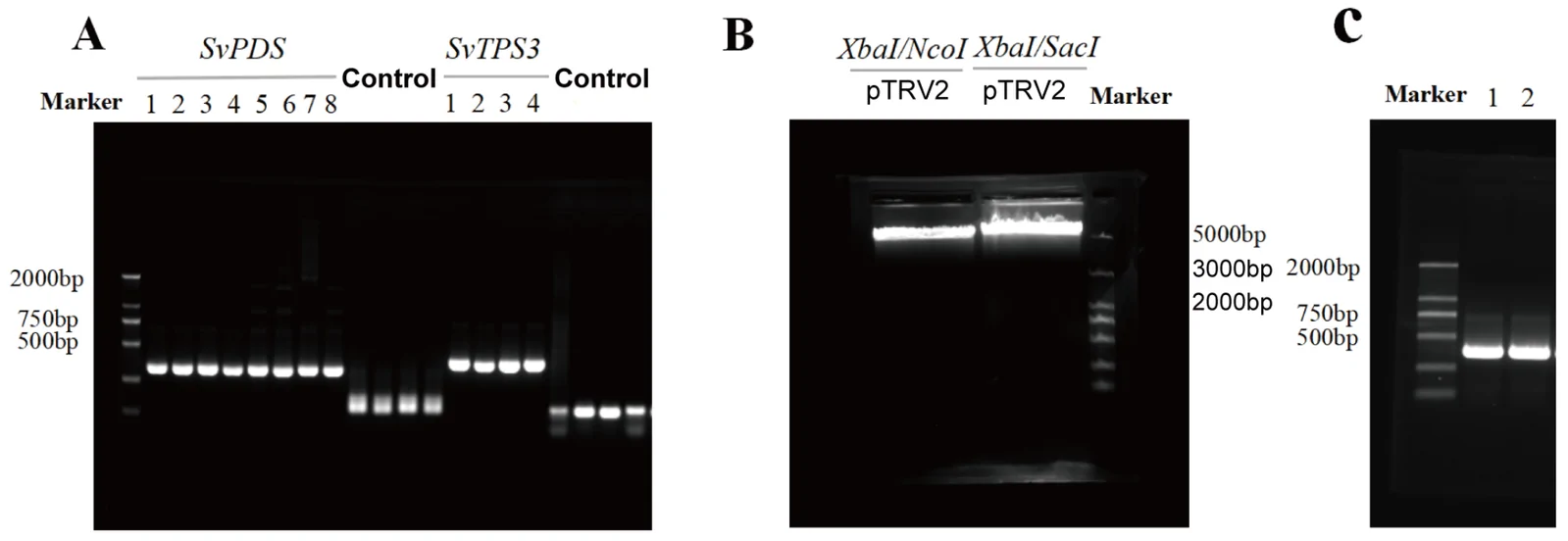
Construction and validation of VIGS vectors for functional analysis in *S. villosa*. (**A**) Amplification of the *SvPDS* and *SvTPS3* target fragments (expected size: 300 bp). (**B**) Restriction digestion analysis of the pTRV2 vector using XbaI/NcoI and XbaI/SacI. The released fragments (12 bp and 34 bp, respectively) were below the resolution of the agarose gel and therefore were not visible. (**C**) PCR identification of recombinant pTRV2-*SvPDS* and pTRV2-*SvTPS3* vectors. A 2000-bp DNA marker was used in (**A**,**C**), with marker sizes indicated on the left, whereas a 5000-bp DNA marker was used in (**B**), with marker sizes indicated on the right.

The *SvPDS* and *SvTPS3* target fragments were successfully amplified, yielding the expected 300-bp products, and the observed bands corresponded to the expected size (Figure 1A). The recombinant pTRV2-*SvPDS* and pTRV2-*SvTPS3* vectors were confirmed by restriction digestion, colony PCR, and sequencing analysis (Figure 1B,C), indicating successful construction of the VIGS vectors used in subsequent experiments.

### 2.2. Optimization of Agrobacterium Concentration for SvPDS Silencing

The concentration of *Agrobacterium* suspension significantly affected *SvPDS* silencing efficiency in *Syringa villosa* seedlings, and the response patterns varied among different inoculation methods (*p* < 0.05; Table 1). Under syringe infiltration conditions, silencing efficiency showed a concentration-dependent response. The highest silencing efficiency was observed at OD_600_ = 0.8 (80.00%), whereas lower efficiencies were detected at OD_600_ = 0.5 (63.64%), 1.0 (35.71%), and 1.5 (42.86%). Although some treatments resulted in a higher number of surviving plants, they did not show greater VIGS efficiency as evaluated by the characteristic photobleaching phenotype induced by *SvPDS* silencing. A similar concentration response was observed in vacuum infiltration. The highest silencing efficiency was obtained at OD_600_ = 0.8 (72.73%), followed by a decrease at higher bacterial concentrations. Root irrigation showed relatively stable but limited silencing efficiency across the tested concentrations, whereas friction inoculation displayed a decreasing trend with increasing bacterial concentration. Overall, OD_600_ = 0.8 showed better performance among the tested concentrations and was selected as the appropriate bacterial concentration for subsequent optimization of the *S. villosa* VIGS system.

### 2.3. Optimization of Inoculation Methods for SvPDS Silencing

Among the tested inoculation methods, syringe infiltration and vacuum infiltration showed higher *SvPDS* silencing efficiencies, whereas root irrigation and friction inoculation resulted in relatively weaker responses. Among the four tested approaches, syringe infiltration and vacuum infiltration showed higher silencing efficiencies, whereas root irrigation and friction inoculation resulted in relatively weaker responses. Using the optimized bacterial concentration (OD_600_ = 0.8), syringe infiltration achieved the highest silencing efficiency (80.00%), followed by vacuum infiltration (72.73%). In comparison, root irrigation and friction inoculation showed lower silencing efficiencies, with maximum values below 50%. Although vacuum infiltration produced a comparable silencing efficiency, syringe infiltration required simpler operation and showed better applicability for subsequent experiments. Therefore, syringe infiltration combined with OD_600_ = 0.8 was selected as the optimized VIGS strategy for functional analysis in *S. villosa*.

### 2.4. Phenotypic and Molecular Validation of SvPDS Silencing in S. villosa

**Figure 2:**
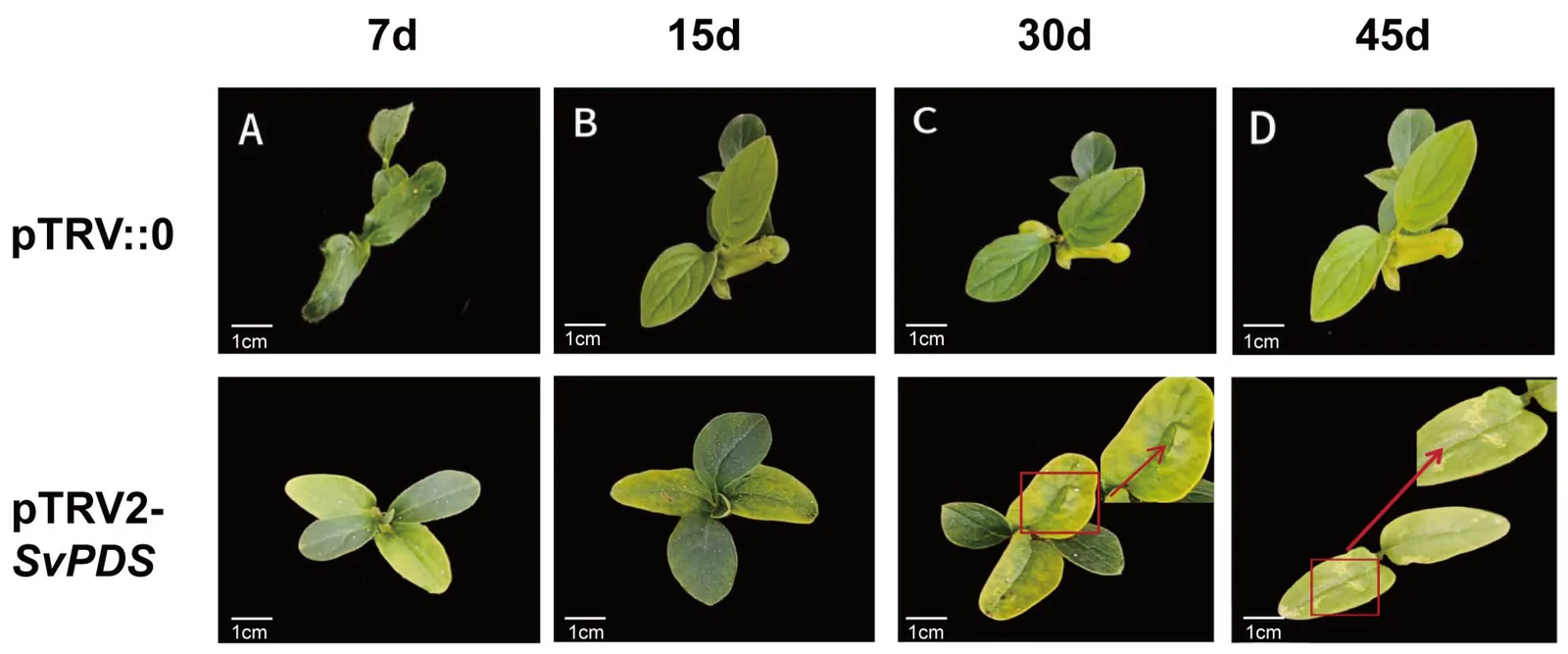
Photobleaching phenotype of *S. villosa* seedlings following SvPDS silencing by syringe infiltration. (**A**) Photographs of pTRV::00- and pTRV2-SvPDS-treated seedlings at 7 dpi. (**B**) Photographs of pTRV::00- and pTRV2-SvPDS-treated seedlings at 15 dpi. (**C**) Photographs of pTRV::00- and pTRV2-SvPDS-treated seedlings at 30 dpi. (**D**) Photographs of pTRV::00- and pTRV2-SvPDS-treated seedlings at 45 dpi. Red boxes and arrows indicate representative photobleached leaf regions. Red boxes indicate the photobleaching phenotype observed in pTRV2-SvPDS-treated seedlings. Scale bars, 1 cm.

**Figure 3:**
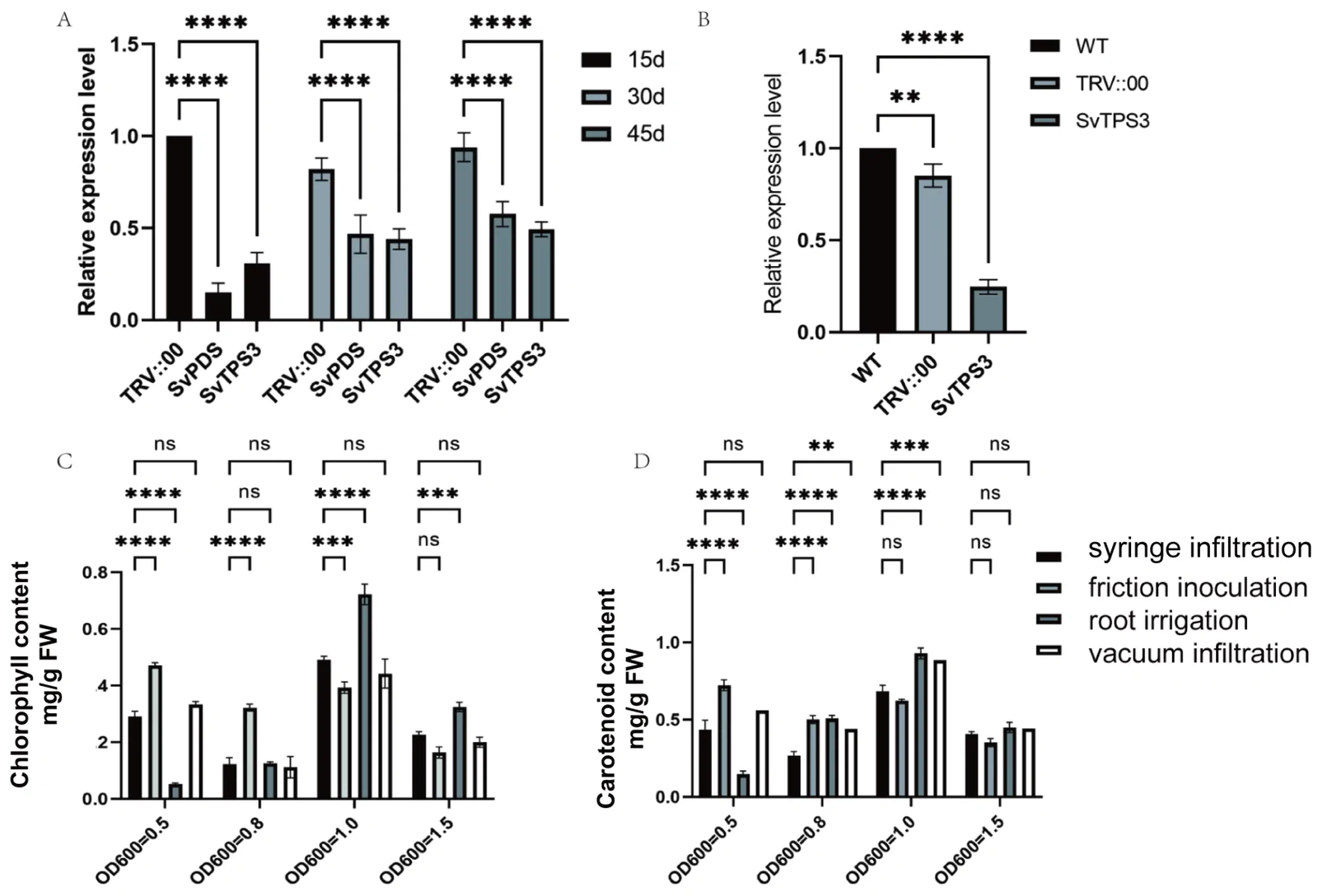
Molecular and physiological validation of VIGS-mediated silencing of *SvPDS* and *SvTPS3* in *S. villosa*. (**A**) Relative expression levels of *SvPDS* and *SvTPS3* following the corresponding VIGS treatments at different days post inoculation (dpi). *SvPDS* and *SvTPS3* were silenced using pTRV2-*SvPDS* and pTRV2-*SvTPS3*, respectively, by syringe infiltration of leaves, with TRV::00 as the empty vector control. (**B**) Relative expression levels of *SvTPS3* in detached petals following *Agrobacterium*-mediated vacuum infiltration with pTRV2-*SvTPS3* or the empty vector control (TRV::00). (**C**) Chlorophyll content in *S. villosa* seedlings subjected to different VIGS inoculation methods and *Agrobacterium* concentrations at 45 dpi. (**D**) Carotenoid content in *S. villosa* seedlings subjected to different VIGS inoculation methods and *Agrobacterium* concentrations at 45 dpi. ** indicates *p* < 0.01, *** indicates *p* < 0.001, **** indicates *p* < 0.0001, and ns indicates no significant difference.

To evaluate the silencing efficiency of the optimized VIGS system, phenotypic observation and qRT-PCR analysis were performed at different time points after inoculation with pTRV2-*SvPDS*. Compared with the empty vector control (pTRV2-TRV::00), plants inoculated with pTRV2-*SvPDS* gradually developed a photobleaching phenotype. Scattered white spots first appeared on leaves at 15 days post inoculation (dpi), while more pronounced photobleaching symptoms were observed at 30 and 45 dpi (Figure 2). qRT-PCR analysis further confirmed the reduction in endogenous *SvPDS* transcripts (Figure 3A). Compared with the control group, *SvPDS* transcript levels were significantly decreased in pTRV2-*SvPDS*-inoculated plants at all examined time points. The strongest suppression was detected at 15 dpi, followed by a slight increase at later stages, although transcript levels remained lower than those in control plants. Notably, the temporal pattern of *SvPDS* transcript suppression did not completely parallel the progression of the photobleaching phenotype, which became more pronounced at 30 and 45 dpi. The strongest suppression was detected at 15 dpi, followed by a slight increase at later stages, although transcript levels remained lower than those in control plants. These results indicated that the optimized VIGS conditions effectively induced *SvPDS* silencing in *S. villosa*. Photosynthetic pigment contents were further evaluated in *S. villosa* seedlings subjected to the different VIGS inoculation conditions at 45 dpi (Figure 3C,D). Chlorophyll and carotenoid contents varied among the different inoculation methods and *Agrobacterium* concentrations, consistent with differences in the photobleaching phenotype observed following *SvPDS* silencing. Together with the phenotypic observations and qRT-PCR results, these findings further supported the effectiveness of the optimized VIGS conditions in *S. villosa*.

### 2.5. Molecular Characterization and Functional Analysis of the Candidate Terpene Synthase Gene SvTPS3

#### 2.5.1. Cloning and Bioinformatic Characterization of SvTPS3

**Figure 4:**
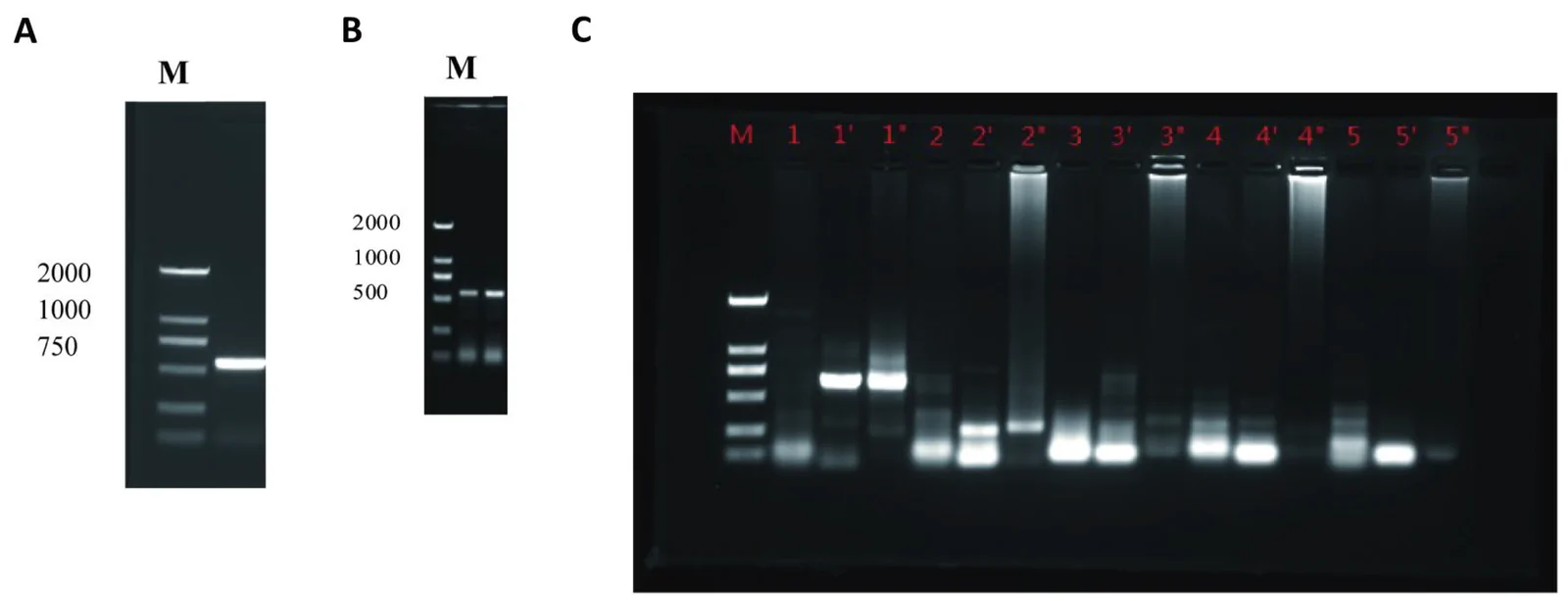
Isolation of the full-length cDNA sequence of *SvTPS3* from *S. villosa*. (**A**) Amplification of the conserved fragment of *SvTPS3* using degenerate primers. (**B**) Amplification of the 3′ end of *SvTPS3* obtained by rapid amplification of cDNA ends (RACE). (**C**) Amplification of the 5′ end of *SvTPS3* obtained by HiTAIL-PCR.

**Figure 5:**
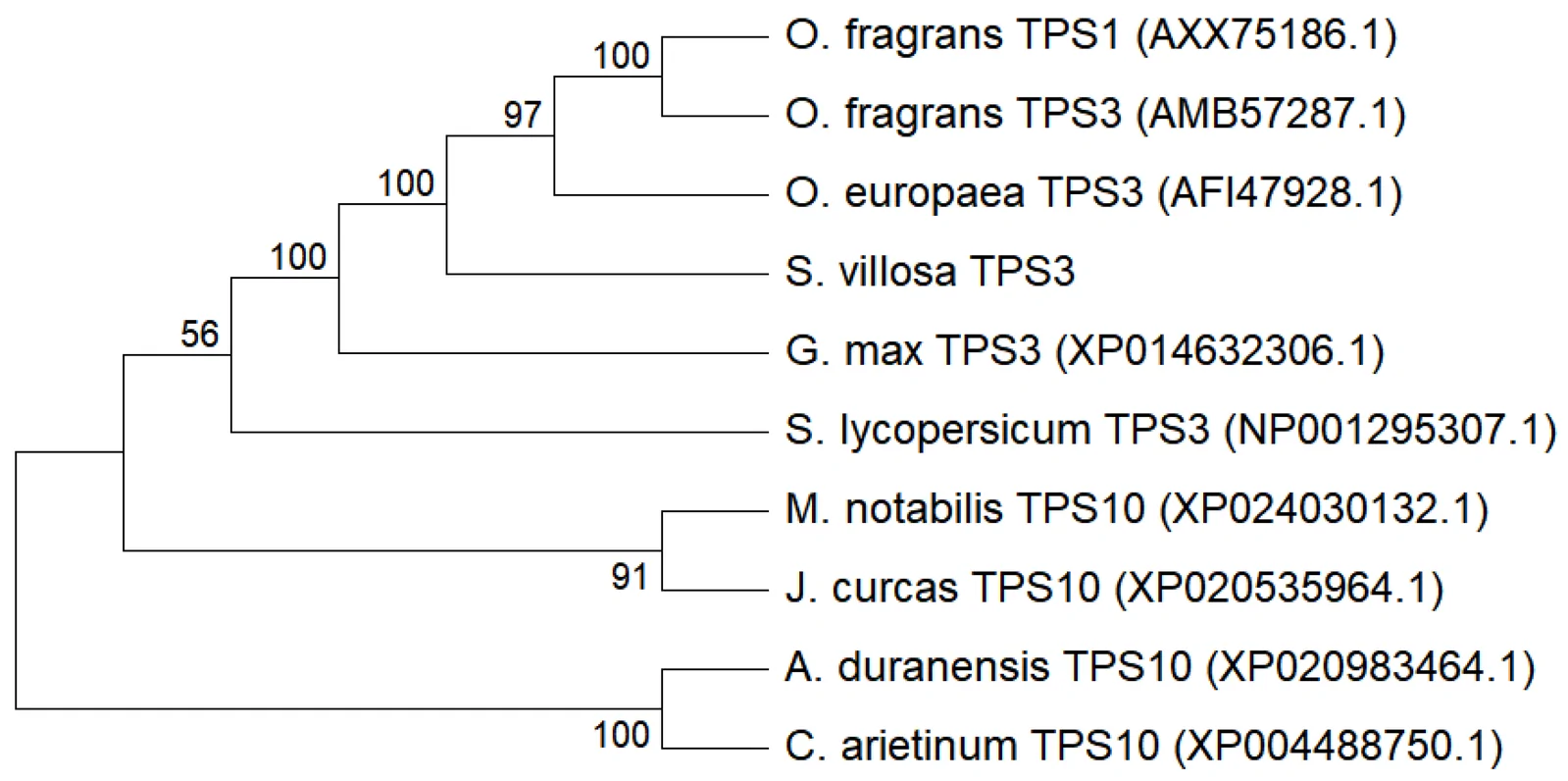
Multiple sequence alignment and phylogenetic analysis of *SvTPS3* and related TPS proteins from different plant species.

To identify terpene synthase candidates potentially associated with floral volatile formation in *S. villosa*, the full-length cDNA sequence of *SvTPS3* was obtained from full-bloom flowers using homologous cloning and RACE amplification (Figure 4). The obtained *SvTPS3* cDNA sequence was 1856 bp in length and contained a complete open reading frame (ORF) of 1776 bp, encoding a predicted protein of 591 amino acids. The nucleotide sequence of *SvTPS3* has been deposited in GenBank under accession number QB133568. Conserved domain analysis indicated that the deduced SvTPS3 protein contained typical characteristics of the plant terpene synthase (TPS) family. Multiple sequence alignment showed that SvTPS3 shared high amino acid sequence similarity with TPS proteins from related species, particularly those from *O. fragrans* and *Olea europaea*. Phylogenetic analysis further grouped SvTPS3 with OfTPS3, OeTPS3, and OfTPS1, with bootstrap support values shown at the corresponding nodes in Figure 5. These results support the placement of SvTPS3 within a TPS subgroup containing monoterpene synthase proteins.

#### 2.5.2. Functional Analysis of SvTPS3 Using the Optimized VIGS System

To further evaluate the applicability of the optimized VIGS system in floral tissues, *SvTPS3* was silenced in petals of *Syringa villosa* at the full-bloom stage. Compared with the empty vector control (TRV::00), petals infiltrated with pTRV2-*SvTPS3* showed a significant reduction in *SvTPS3* transcript levels, as determined by qRT-PCR analysis (Figure 3B).

**Figure 6:**
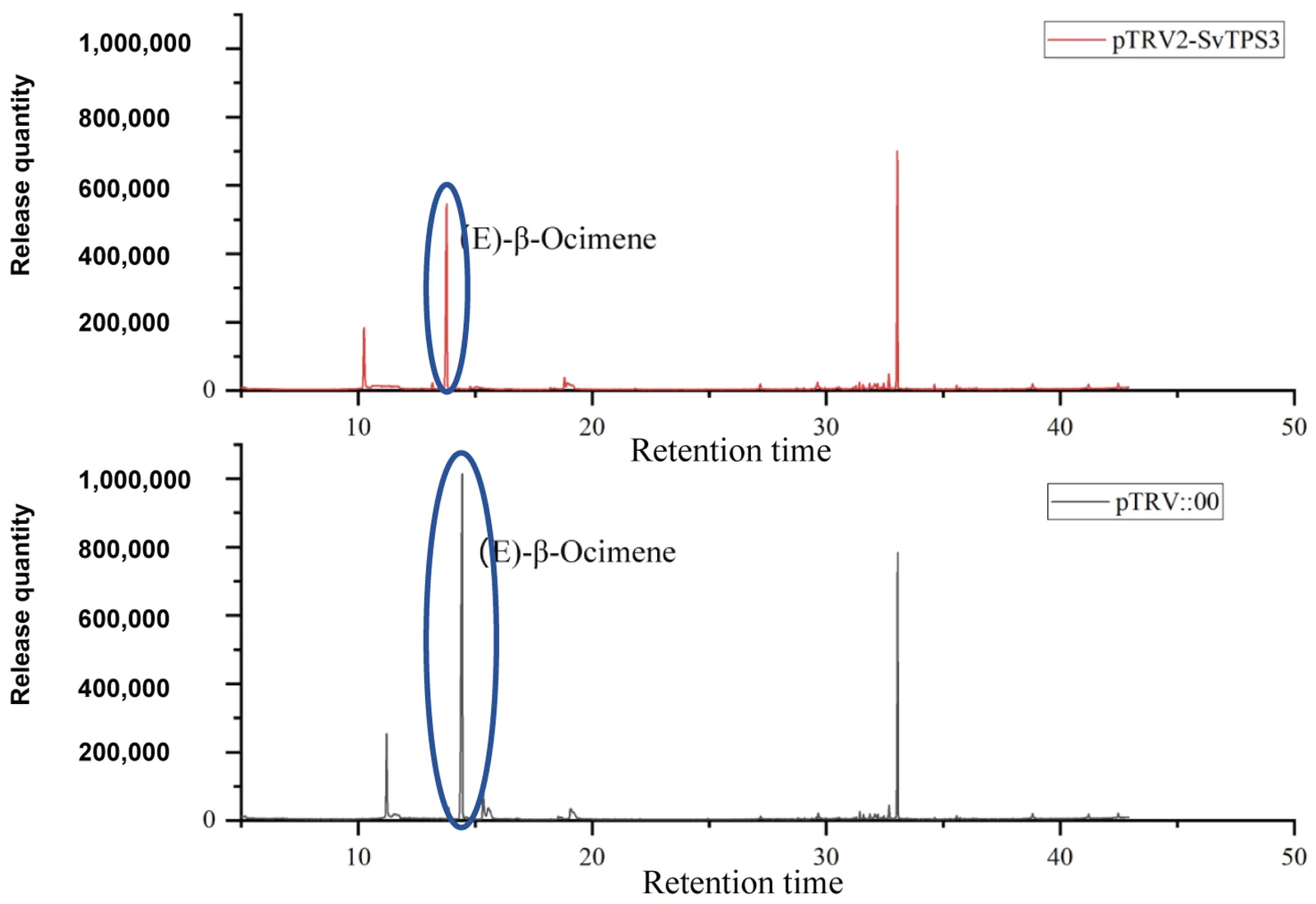
Representative GC-MS chromatograms of volatile compounds in *S. villosa* petals following VIGS treatment. The upper and lower panels show representative chromatograms of pTRV2-*SvTPS3*-treated and TRV::00 control petals, respectively. The labeled peak indicates the chromatographic peak annotated as (E)-β-ocimene.

**Figure 7:**
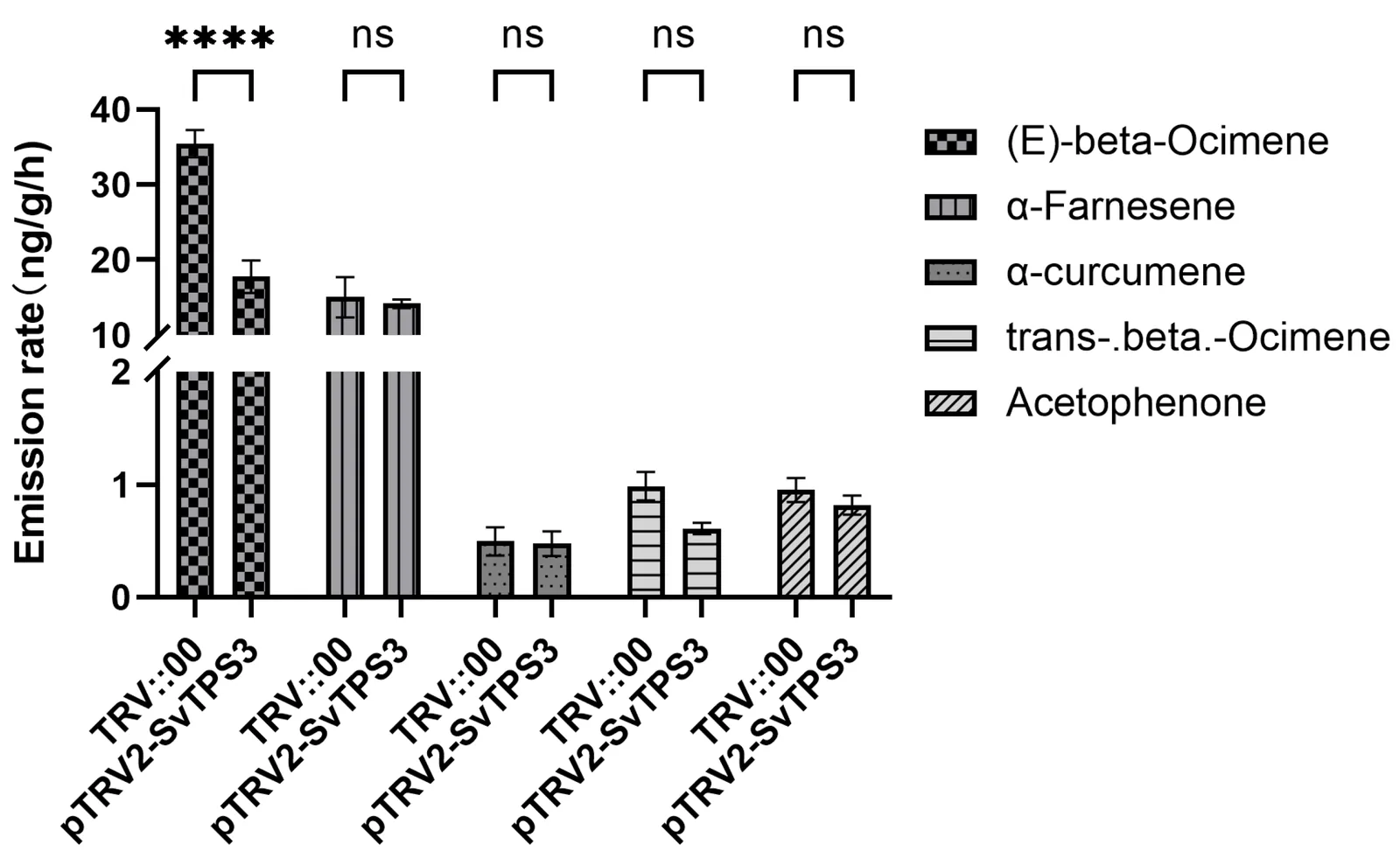
Changes in major floral volatile compounds after *SvTPS3* silencing in *S. villosa*. **** indicates *p* < 0.0001, and ns indicates no significant difference.

Consistent with the decrease in *SvTPS3* transcript abundance, the emission rate of (E)-β-ocimene, the predominant volatile compound detected in *S. villosa* flowers, was significantly reduced in *SvTPS3*-silenced petals, reaching approximately 50% of the control level (*p* < 0.0001) (Figure 6 and Figure 7). In contrast, the emission rates of other major volatile compounds, including α-farnesene, α-curcumene, trans-β-ocimene, and acetophenone, showed no significant differences between the pTRV2-*SvTPS3* and control groups (*p* > 0.05).

These results indicate that the optimized VIGS system established in *S. villosa* can be applied to floral tissues for gene function analysis. The reduced accumulation of (E)-β-ocimene following *SvTPS3* silencing suggests that *SvTPS3* contributes to the regulation of (E)-β-ocimene accumulation in *S. villosa* flowers.

## 3. Discussion

Woody plants generally have long generation cycles and are often difficult to genetically transform, which restricts functional genomic studies of important traits [34,35]. *S. villosa* is an important ornamental woody species with valuable floral traits, including flower color and fragrance; however, the genes and metabolic pathways underlying these traits remain poorly characterized due to the lack of efficient genetic manipulation tools. Although VIGS has been applied in several *Syringa* species, an optimized VIGS system specifically for *S. villosa* has not been reported. In this study, we established an *Agrobacterium*-mediated VIGS system in *S. villosa*, providing a transient and efficient approach for functional analysis of candidate genes associated with ornamental traits.

The efficiency of VIGS is affected by multiple factors, including inoculation method, bacterial concentration, host characteristics, and virus–plant compatibility [29,36,37]. Different plant species often require optimization of inoculation procedures because tissue structure and infection efficiency vary among hosts [38]. In the present study, syringe infiltration and vacuum infiltration showed higher *SvPDS* silencing efficiencies than root irrigation and friction inoculation. Similar differences among inoculation methods have been reported in other species, indicating that optimization of delivery strategies is essential for establishing effective VIGS systems [36,39].

*Agrobacterium* concentration is another critical factor influencing VIGS efficiency. Previous studies have demonstrated that appropriate bacterial density promotes viral delivery and gene silencing, whereas excessive bacterial concentrations may cause tissue damage and negatively affect plant growth [33,39,40]. In *S. villosa*, gene silencing was successfully induced within the tested OD_600_ range of 0.5–1.5, with OD_600_ = 0.8 providing the highest silencing efficiency under the tested conditions. As a woody species, *S. villosa* may differ from commonly used herbaceous VIGS hosts in its response to *Agrobacterium*-mediated VIGS, and VIGS conditions often require host-specific optimization [29,35]. Such host-specific differences may partly explain why a relatively higher bacterial density was more effective in the present study. Under our experimental conditions, an OD_600_ of 0.8 may have provided a more effective bacterial inoculum for VIGS induction than the lower concentrations tested, whereas further increases in bacterial density did not improve silencing efficiency. Similar concentration-dependent effects have been reported in other species, although optimal bacterial densities differ among plant–virus systems [33].

Similar VIGS approaches have also been successfully applied in other ornamental plants. For example, a TRV-based VIGS system was developed in Hydrangea macrophylla and used to characterize the function of HmF3′5′H in flower color formation [26]. In tree peony, a VIGS system targeting the flavonoid glycosyltransferase genes PsUF3GT and PsUF5GT was established for functional analysis of genes associated with anthocyanin metabolism and flower coloration [30]. In Forsythia, an efficient VIGS system was established and validated using PDS as a reporter gene [29]. In Senecio cruentus, VIGS-mediated silencing of ScbHLH17 revealed its involvement in anthocyanin biosynthesis [31]. Collectively, these studies demonstrate the utility of VIGS for rapid functional analysis of genes associated with ornamental traits and specialized metabolism across diverse ornamental species and, together with the present results in *S. villosa*, support its applicability as a functional genomics tool in ornamental plants [26,29,30,31].

It should be noted that the temporal pattern of the photobleaching phenotype did not completely parallel the changes in *SvPDS* transcript abundance. The strongest reduction in *SvPDS* transcript abundance was detected at 15 dpi, whereas the photobleaching phenotype became more pronounced at 30 and 45 dpi. Although *SvPDS* transcript abundance partially recovered after 15 dpi, it remained lower than that in the control plants at the later time points. This apparent discrepancy may reflect a temporal lag between transcript-level silencing and the development of visible phenotypic effects. qRT-PCR reflects transcript abundance at a specific sampling time, whereas photobleaching represents a downstream physiological consequence of impaired PDS function that may progressively develop following transcriptional suppression. Thus, the effects initiated during the period of stronger *SvPDS* suppression may persist and contribute to the progressive development of photobleaching even when transcript abundance subsequently increases. Therefore, the most pronounced visible phenotype may not necessarily coincide with the time point showing the strongest reduction in transcript abundance. In addition, the photobleaching phenotype induced by *SvPDS* silencing is associated with disruption of carotenoid biosynthesis and may be accompanied by secondary physiological effects and broader transcriptional reprogramming. Therefore, phenotypic changes observed during *SvPDS*-based evaluation of VIGS efficiency should be interpreted with caution, as they may reflect secondary physiological responses in addition to target-specific gene silencing. In future studies, combining VIGS with transcriptome analysis or using alternative reporter genes with milder phenotypes may help distinguish target-specific effects from broader physiological responses.

Terpene synthases represent a major enzyme family responsible for generating structural diversity of plant volatile terpenoids and play important roles in shaping species-specific floral scent profiles. Monoterpenes, including (E)-β-ocimene, are widely distributed floral volatile compounds that contribute to plant–pollinator interactions and floral aroma characteristics. Previous studies have demonstrated that TPS genes are involved in β-ocimene formation in several ornamental plants. For example, OfTPS3 and OfTPS1 from *O. fragrans* have been associated with β-ocimene and β-linalool production, respectively, while TPS genes from *Syringa oblata* have also been implicated in ocimene biosynthesis [8,9,10,11,12].

In this study, the full-length sequence of *SvTPS3* was obtained from *S. villosa* flowers, and sequence analysis showed that it clustered with TPS proteins involved in monoterpene formation. Application of the optimized VIGS system to floral tissues resulted in a significant reduction in *SvTPS3* transcript abundance and a corresponding decrease in the emission rate of (E)-β-ocimene. The specific reduction in (E)-β-ocimene, without significant changes in other major volatile compounds, suggests that *SvTPS3* may preferentially contribute to the regulation of (E)-β-ocimene accumulation in *S. villosa* flowers. However, biochemical assays, including heterologous expression and enzyme activity analysis, are still required to confirm the catalytic function and substrate specificity of *SvTPS3*.

A notable contribution of this study is the integration of VIGS system optimization with its application to floral scent-related gene analysis in *S. villosa*. Beyond establishing suitable conditions for VIGS in vegetative tissues, the optimized system was successfully extended to detached floral tissues, demonstrating its applicability to the rapid functional analysis of genes associated with floral traits and specialized metabolism. The application of this system to *SvTPS3* further provides functional evidence linking this candidate TPS gene to (E)-β-ocimene accumulation. Thus, the established VIGS platform expands the experimental tools available for functional genomic studies of floral traits in *S. villosa*.

Although the VIGS system established in this study provides an effective tool for functional analysis in *S. villosa*, several limitations remain. As a transient gene suppression approach, VIGS efficiency may vary among tissues, developmental stages, and target genes. In addition, the present study mainly focused on the association between *SvTPS3* expression and floral volatile changes. Future studies integrating stable transformation, biochemical characterization, and multi-omics approaches will further elucidate the regulatory mechanisms underlying floral scent formation in *Syringa* species.

## 4. Materials and Methods

### 4.1. Plant Materials and Growth Conditions

Seeds of *S. villosa* were collected from the No. 1 Experimental Station of Hebei Agricultural University. After soaking in water for 24 h, seeds were sown in plug trays containing a mixture of peat, vermiculite, and perlite (3:1:1, *v*/*v*). Seedlings were grown in an artificial climate chamber under controlled conditions of 24 °C, 70% relative humidity, and a 16 h light/8 h dark photoperiod. Four-week-old seedlings were used for the establishment and optimization of the VIGS system. *E.coli* DH5α and *Agrobacterium tumefaciens* GV3101, together with the VIGS vectors used in this study, were maintained in our laboratory. For floral tissue experiments, flowers of *S. villosa* at the full-bloom stage were collected from the same experimental population and used for subsequent *SvTPS3* silencing and floral volatile analysis.

### 4.2. Amplification of SvPDS and SvTPS3 Fragments and Construction of VIGS Vectors

To establish the VIGS system in *S. villosa*, *SvPDS* was selected as the reporter gene. In addition, the candidate terpene synthase gene *SvTPS3*, which was potentially associated with floral volatile formation, was selected for subsequent functional analysis. The *SvPDS* sequence was obtained based on transcriptome information of *S. villosa* leaves, whereas the full-length sequence of *SvTPS3* was obtained through homologous cloning and RACE approaches. Total RNA was extracted from plant tissues using the CWBIO RNA extraction kit (CW0535; CWBIO, Beijing, China). RNA integrity was evaluated by 1.0% agarose gel electrophoresis, and RNA concentration was determined using a NanoDrop 2000 spectrophotometer (Thermo Fisher Scientific, Waltham, MA, USA). High-quality RNA was reverse-transcribed into cDNA using the PrimeScript™ FAST RT Reagent Kit with gDNA Eraser (TaKaRa, Kusatsu, Japan) according to the manufacturer’s instructions.

VIGS target fragments were designed according to the general principles of VIGS fragment selection, with reference to the SGN-VIGS tool. Because genomic sequence information of *S. villosa* is not available in the SGN-VIGS database, the target sequences were obtained from *S. villosa*-specific sequence resources, including transcriptomic data for *SvPDS* and cloned full-length cDNA sequences for *SvTPS3*. Candidate fragments were selected based on sequence specificity and suitability for VIGS-mediated silencing. The selected VIGS target fragments of *SvPDS* and *SvTPS3* were both 300 bp in length, and their complete sequences are provided in Supplementary File S1. Specific primers were designed using DNAMAN and Primer Premier 5 software and synthesized by Sangon Biotech (Shanghai, China) (Table 2). The VIGS system used in this study was based on the bipartite tobacco rattle virus (TRV) vector system consisting of pTRV1 and pTRV2 vectors. The empty pTRV2 vector (TRV::00) was used as the negative control, whereas gene-specific fragments of *SvPDS* and *SvTPS3* were inserted into the pTRV2 vector to generate pTRV2-*SvPDS* and pTRV2-*SvTPS3* constructs, respectively. The pTRV2 vector was linearized by double digestion with FastDigest restriction enzymes (XbaI/NcoI or XbaI/SacI; Takara, Kusatsu, Japan). The corresponding restriction sites and the 12-bp and 34-bp fragments released by XbaI/NcoI and XbaI/SacI digestion, respectively, are shown in Supplementary Figure S1. Target fragments of *SvPDS* and *SvTPS3* were amplified from *S. villosa* cDNA using 2× Super Pfx Master Mix (CW0535; CWBIO, Beijing, China), and the corresponding restriction sites were introduced into the primers. The amplified fragments were purified and inserted into the linearized pTRV2 vector using the Minerva Super Fusion Cloning Kit (US Everbright^®^ Inc., Suzhou, China). The recombinant plasmids were transformed into *Escherichia.coli* DH5α competent cells and screened by colony PCR. Positive clones were verified by sequencing at Sangon Biotech (Shanghai, China). Schematic maps of the confirmed recombinant pTRV2-*SvPDS* and pTRV2-*SvTPS3* vectors are provided in Supplementary Figure S2. The confirmed VIGS vectors pTRV2-*SvPDS* and pTRV2-*SvTPS3* were subsequently transformed into *Agrobacterium tumefaciens* GV3101 for VIGS experiments.

### 4.3. Agrobacterium-Mediated Establishment and Optimization of the VIGS System

The VIGS vectors were transformed into *A. tumefaciens* GV3101 competent cells using the freeze–thaw method. Positive transformants were selected on solid LB medium supplemented with kanamycin (25 μg/mL) and rifampicin (25 μg/mL) and incubated at 28 °C for 3–4 days. A single positive colony was subsequently inoculated into 10 mL of LB liquid medium containing kanamycin (25 μg/mL) and rifampicin (25 μg/mL) and cultured at 28 °C with shaking at 200 rpm for 24 h. Subsequently, 1 mL of the bacterial culture was transferred into 100 mL of LB liquid medium containing the same antibiotics and cultured at 28 °C with shaking at 200 rpm for an additional 10 h. The bacterial cells were collected by centrifugation at 4000 rpm at 4 °C and resuspended in infiltration solution consisting of MS medium supplemented with 10 mM MES, 10 mM MgCl_2_, and 200 μM acetosyringone. The bacterial suspensions were adjusted to the required OD_600_ values and incubated in darkness at room temperature for 3 h. Immediately before inoculation, equal volumes of the pTRV1 suspension and the corresponding pTRV2, pTRV2-*SvPDS*, or pTRV2-*SvTPS3* suspension were mixed at a 1:1 (*v*/*v*) ratio. Four-week-old *Syringa villosa* seedlings were used for VIGS optimization. Four inoculation methods, including syringe infiltration, friction inoculation, root irrigation, and vacuum infiltration, were evaluated under different *Agrobacterium* concentrations (OD_600_ = 0.5, 0.8, 1.0, and 1.5). For syringe infiltration, a small puncture was first made on the abaxial surface of each leaf using a sterile syringe needle. A needleless syringe containing the bacterial suspension was then gently pressed against the puncture site, and the suspension was slowly infiltrated into the leaf tissue. A total of 50 mL of bacterial suspension was used to treat 50 seedlings for each syringe infiltration treatment. For friction inoculation, leaves were gently rubbed with quartz sand coated with bacterial suspension to generate micro-wounds for *Agrobacterium* infection. A total of 50 mL of bacterial suspension was used to treat 50 seedlings for each friction inoculation treatment. For root irrigation, bacterial suspension was directly applied to the root system. A total of 50 mL of bacterial suspension was applied to the root systems of 50 seedlings for each root irrigation treatment. For vacuum infiltration, seedlings were immersed in bacterial suspension and treated under vacuum for 10 min, followed by gradual restoration of atmospheric pressure. After inoculation, all treated plants were maintained in darkness for 24 h and subsequently transferred to normal growth conditions. Each treatment included three independent biological replicates.

### 4.4. Application of the Optimized VIGS System to Floral Tissues

Based on the optimized VIGS conditions established in *S. villosa* seedlings, the *Agrobacterium*-mediated infiltration procedure was further applied to floral tissues for functional analysis of *SvTPS3*. Petals from flowers at the full-bloom stage were collected and immersed in *Agrobacterium* suspension containing pTRV1/pTRV2-*SvTPS3* or pTRV1/pTRV2 empty vector at a 1:1 ratio. The bacterial suspension was adjusted to OD_600_ = 0.8 according to the optimized conditions obtained from leaf experiments. Petal tissues were subjected to vacuum infiltration for 10 min. Following infiltration, the petals were washed 4–5 times with 5% (*w*/*v*) sucrose solution to remove residual bacterial suspension. The washed, detached petals were then completely immersed in fresh 5% (*w*/*v*) sucrose solution and maintained at 26 °C under a 12 h light/12 h dark photoperiod for 24 h. The petals were subsequently collected for qRT-PCR analysis and volatile compound determination. The sampling time was determined based on preliminary observations of petals at 24, 36, 48, and 72 h after infiltration. At 24 h, the petals retained a fresh appearance and their characteristic floral scent. Changes in floral scent became noticeable at 36 h, whereas yellowing of the petal margins and an overall decline in tissue freshness were observed after 48 h. Therefore, 24 h after infiltration was selected as the sampling time point to minimize potential changes in volatile composition associated with deterioration of the detached floral tissues while maintaining the physiological condition of the petals for subsequent gene expression and volatile compound analyses.

### 4.5. Evaluation of VIGS Efficiency

The silencing efficiency of *SvPDS* was evaluated based on the visible photobleaching phenotype. After infiltration, seedlings were monitored every three days, and the number of plants exhibiting the characteristic photobleaching phenotype was recorded at 30 days post infiltration (dpi). The reduction in *SvPDS* transcript abundance was further determined by qRT-PCR to confirm gene silencing at the molecular level.

The silencing efficiency was calculated according to the following formula:

Silencing efficiency (%) = (Number of plants exhibiting the characteristic photobleaching phenotype/Total number of inoculated plants) × 100%.

### 4.6. Determination of Chlorophyll and Carotenoid Contents

Chlorophyll and carotenoid contents were determined according to a previously described method with slight modifications [41]. Leaf samples were collected at 45 days after treatment. Approximately 0.1 g of fresh leaves was weighed, cut into small pieces, and placed into glass tubes containing 10 mL of 95% ethanol. Samples were incubated in darkness for 24 h until complete pigment extraction.

The absorbance values were measured at 665 nm, 649 nm, and 470 nm using a spectrophotometer. Chlorophyll a (Ca), chlorophyll b (Cb), carotenoid content (Cx+c), and total chlorophyll content were calculated using the following equations:

Ca = 13.95A665 − 6.88A649

Cb = 24.96A649 − 7.32A665

Cx’c(carotenoids) = (1000A_470_ − 2.05Ca − 114.8Cb)/245

Total chlorophyll concentration (Ctotal, mg/L) = Ca + Cb

Chlorophyll content (mg/g) = (Ctotal × extraction solution volume)/(sample fresh weight × 1000).

Three independent biological replicates were performed for each treatment.

### 4.7. Gene Expression Analysis

Total RNA was extracted from treated tissues, and first-strand cDNA was synthesized for quantitative real-time PCR (qRT-PCR) analysis. Specific primers for *SvPDS* and *SvTPS3* were designed based on the corresponding sequences of *S. villosa* (Table 2). qRT-PCR was performed using SvGAPDH as the internal reference gene. Relative transcript levels were calculated using the 2^−ΔΔCt^ method. Three independent biological replicates were analyzed for each sample.

### 4.8. Sequence and Phylogenetic Analysis of SvTPS3

The deduced amino acid sequence of SvTPS3 and nine TPS protein sequences from other plant species were used for phylogenetic analysis. The sequences included in the analysis were *O. fragrans* TPS3 (AMB57287.1), *O. fragrans* TPS1 (TPS1AXX75186.1), *O. europaea* TPS3 (AFI47928.1), *S. villosa* TPS3, *Glycine max* TPS3 (XP_014632306.1), *Solanum lycopersicum* TPS3 (NP_001295307.1), *Morus notabilis* TPS10 (XP_024030132.1), *Jatropha curcas* TPS10 (XP_020535964.1), *Cicer arietinum* TPS10 (XP_004488750.1), and *Arachis duranensis* TPS10 (XP_020983464.1). Multiple sequence alignment was performed using ClustalW in MEGA12 (MEGA Software). The phylogenetic tree was constructed using the Neighbor-Joining method, and evolutionary distances were calculated using the p-distance method. The reliability of the tree topology was evaluated using 1000 bootstrap replicates. Positions with less than 95% site coverage were eliminated using the partial deletion option.

### 4.9. Analysis of Floral Volatile Compounds by HS-SPME-GC-MS

Floral volatile compounds were extracted using headspace solid-phase microextraction (HS-SPME). Petals of *Syringa villosa* after VIGS treatment were collected, and 1.0 g (±0.001 g) of fresh sample was placed into a 20 mL headspace vial. A fixed amount of ethyl decanoate was added as an internal standard. Samples were equilibrated in a water bath at 42 °C, and an aged SPME fiber (Supelco, USA) was inserted into the headspace approximately 1 cm above the sample for 40 min to extract volatile compounds. After extraction, the SPME fiber was immediately inserted into the GC injection port and desorbed at 250 °C for 6 min. Each sample was analyzed by GC-MS with three independent biological replicates.

The GC conditions were as follows: the injector temperature was set at 250 °C. The initial oven temperature was maintained at 50 °C, increased to 100 °C at 3 °C/min and held for 2 min, then increased to 200 °C at 6 °C/min, followed by an increase to 250 °C at 10 °C/min and maintained for 10 min. Helium was used as the carrier gas at a flow rate of 1.45 mL/min. The MS conditions were as follows: electron ionization (EI) mode at 70 eV, ion source temperature of 230 °C, transfer line temperature of 250 °C, and scanning range of *m*/*z* 33–450.

Volatile compounds were identified by comparing mass spectra with the NIST library database and referring to reported retention indices. Emission rates of volatile compounds were calculated using the peak area normalization method with ethyl decanoate as the internal standard.

### 4.10. Statistical Analysis

All experimental data were presented as mean ± standard deviation (SD) from at least three independent biological replicates. Statistical analyses were performed using IBM SPSS Statistics 25. One-way analysis of variance (ANOVA) followed by Duncan’s multiple range test was used to determine significant differences among multiple groups, with *p* < 0.05 considered statistically significant. For comparisons between two groups, Student’s *t*-test was applied where appropriate. Data visualization and figure preparation were performed using GraphPad Prism 9 and Adobe Illustrator.

## 5. Conclusions

In this study, an *Agrobacterium*-mediated VIGS system was established and optimized for *S. villosa* using *SvPDS* as a reporter gene. By comparing different inoculation methods and *Agrobacterium* concentrations, syringe infiltration with an *Agrobacterium* suspension at an OD_600_ of 0.8 was identified as a suitable condition for efficient gene silencing. The optimized VIGS system was successfully applied not only in vegetative tissues but also in floral tissues of *S. villosa*. Furthermore, the function of the candidate terpene synthase gene *SvTPS3* was evaluated using this platform, and silencing of *SvTPS3* resulted in reduced transcript abundance and decreased accumulation of (E)-β-ocimene in flowers. The VIGS system established in *S. villosa* provides an effective tool for functional genomics studies and facilitates the investigation of genes involved in floral traits and secondary metabolism in *Syringa* species.

## 6. Patents

A Method for Virus-Induced Gene Silencing in *S. villosa*. Chinese Patent Application No. 202610551687.0 (pending).

## Figures and Tables

**Table 1:** Effects of *Agrobacterium* concentration and inoculation method on *SvPDS* silencing efficiency in *Syringa villosa*.

Infection Methods	*Agrobacterium* Concentration (OD_600_)	Number of Surviving Plants	Silenced Plants	Silencing Efficiency
syringe infiltration	0.5	44	28	63.64%
	0.8	40	32	80.00%
	1.0	56	20	35.71%
	1.5	56	24	42.86%
root irrigation	0.5	68	16	23.53%
	0.8	36	12	33.33%
	1.0	42	14	33.33%
	1.5	42	6	14.29%
friction inoculation	0.5	60	28	46.67%
	0.8	30	8	26.67%
	1.0	40	10	25.00%
	1.5	40	10	25.00%
vacuum infiltration	0.5	45	29	64.44%
	0.8	44	32	72.73%
	1.0	48	19	39.58%
	1.5	52	8	15.38%

**Table 2:** Primer sequences used for gene cloning, VIGS vector construction, and qRT-PCR analysis in *S. villosa*.

Function	Primer Name	Primer Sequence (5′-3′)
Vector construction	pTRV2-*SvPDS*-F	TGAGTAAGGTTACCGAATTCTCTAGAGGCAATCCACCAGAAAGACTTTGC
	pTRV2-*SvPDS*-R	GTGAGCTCGGTACCGGATCCCCATGGACAGACATGTCAGCATACACACTGAG
	pTRV2-*SvTPS3*-F	TGAGTAAGGTTACCGAATTCTCTAGATATTGAAGCATACAGTAAAAAAGATG
	pTRV2-*SvTPS3*-R	GCCCGGGCCTCGAGACGCGTGAGCTCTCAAGAGAGCCATAGACATCAT
Carrier verification	pTRV1-F	GCTCTTGGGAACTACATGGTG
	pTRV1-R	CGCCTCAATCGTCTTCATCTCC
	pTRV2-F	GGTACGTAGTAGAGTCCCAC
	pTRV2-R	ACAAAAGACTTACCGATCAATC
qRT-PCR	pTRV2-*SvPDS*-qF	TGGCTGGTTTGTCTACTG
	pTRV2-*SvPDS*-qR	GCCTATCGTTGATGCCTA
	pTRV2-*SvTPS3*-qF	TAAGGAACAAGGTCAGGTCATC
	pTRV2-*SvTPS3*-qR	AGCCATCCACTACTGAGATACTC
Actin gene	*SvGAPDH*-F	CCACTCTATCACGGCTACTCAA
	*SvGAPDH*-R	CAACCACGGAGACATCAACAG
*SvPDS* CDS sequence	*SvPDS*-F	ATGTCCCAAT TTGGACATGT TTCTGCTG
	*SvPDS*-R	TTAAACAAGGCTTGCCTCGGTCGAC
*SvTPS3* CDS sequence	*SvTPS3*-F1	GAG/TCTCCAAC/GAAATGTT/CAAGGTGGTGG
	*SvTPS3*-R1	ATTGAG/AC/TTTGCT/CGT/C/ATTCACCTCT
	*SvTPS3*-3′F	GCTATCATCATCTGGACCACTTCTTC
	B26	GAGTCTAGACGACATCGATTTTTTTTTTTTTTT
	LAD1-1	ACGATGGACTCCAGAGCGGCCGC(G/C/A)N(G/C/A)NNNGGAA
	AC1	ACGATGGACTCCAGAG
	TR-1	GTTCAAATACCATTCCAACTGACCAAAAGAAGCATT
	TR-2	GTTCTTCAAGAGAGCCATAGACATCATAAACATCGTC
	TR-3	GAACAAGAAGAAGTGGTCCAGATGATGATAG

## Data Availability

Data will be made available upon request.

## Supplementary Materials

The following supporting information can be downloaded at https://www.mdpi.com/article/10.3390/plants15203179/s1. Supplementary File S1. VIGS target fragments of SvPDS and SvTPS3 (300 bp each); Supplementary Figure S1. Restriction map of the pTRV2 vector showing the restriction sites used for VIGS vector construction. The distances between the XbaI and NcoI sites and between the XbaI and SacI sites are 12 bp and 34 bp, respectively. Supplementary Figure S2A. Schematic map of the recombinant pTRV2-*SvPDS* vector. Schematic representation of the recombinant pTRV2-*SvPDS* vector constructed for VIGS-mediated silencing of *SvPDS* in *S. villosa*; Supplementary Figure S2B. Schematic map of the recombinant pTRV2-*SvTPS3* vector. Schematic representation of the recombinant pTRV2-SvTPS3 vector constructed for VIGS-mediated silencing of SvTPS3 in *S. villosa*.
