# Supplementary material for: Establishment of a Virus-Induced Gene Silencing (VIGS) System in Syringa villosa and Functional Characterization of the Floral Scent-Associated Gene SvTPS3
Source: Plants (Basel). 2026 Oct 16;15(20):3179. doi: 10.3390/plants15203179 (PMC13709108; doi:10.3390/plants15203179)
Supplement: Supplementary file 1 [file plants-15-03179-s001.zip › Supplementary Figure S1.pdf]

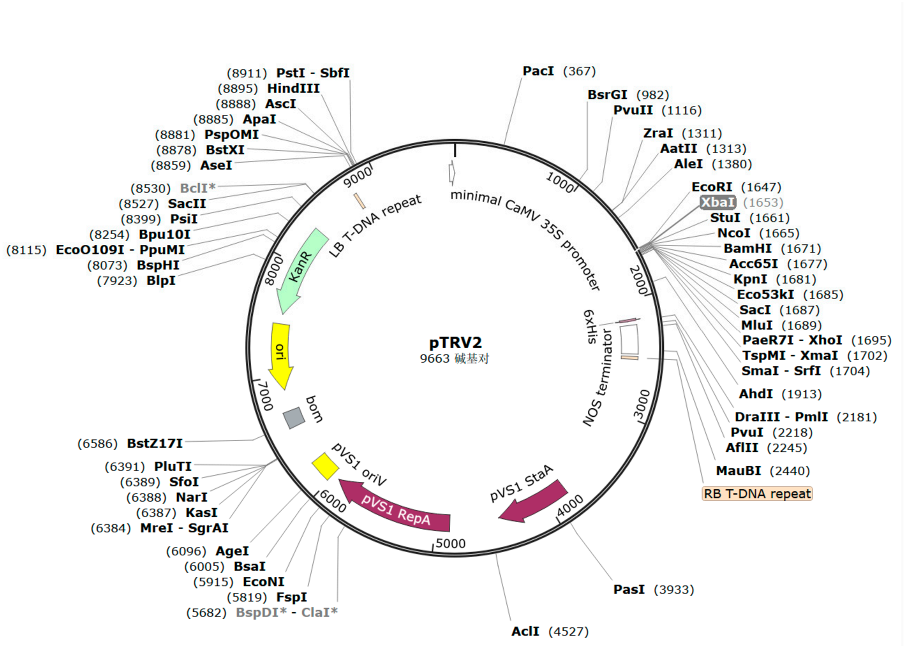

Supplementary Figure S1. Restriction map of the pTRV2 vector showing the restriction sites used for VIGS vector construction. The distances between the XbaI and NcoI sites and between the XbaI and SacI sites are 12 bp and 34 bp, respectively.
