# Supplementary material for: Establishment of a Virus-Induced Gene Silencing (VIGS) System in Syringa villosa and Functional Characterization of the Floral Scent-Associated Gene SvTPS3
Source: Plants (Basel). 2026 Oct 16;15(20):3179. doi: 10.3390/plants15203179 (PMC13709108; doi:10.3390/plants15203179)
Supplement: Supplementary file 1 [file plants-15-03179-s001.zip › Supplementary Figure S2.pdf]

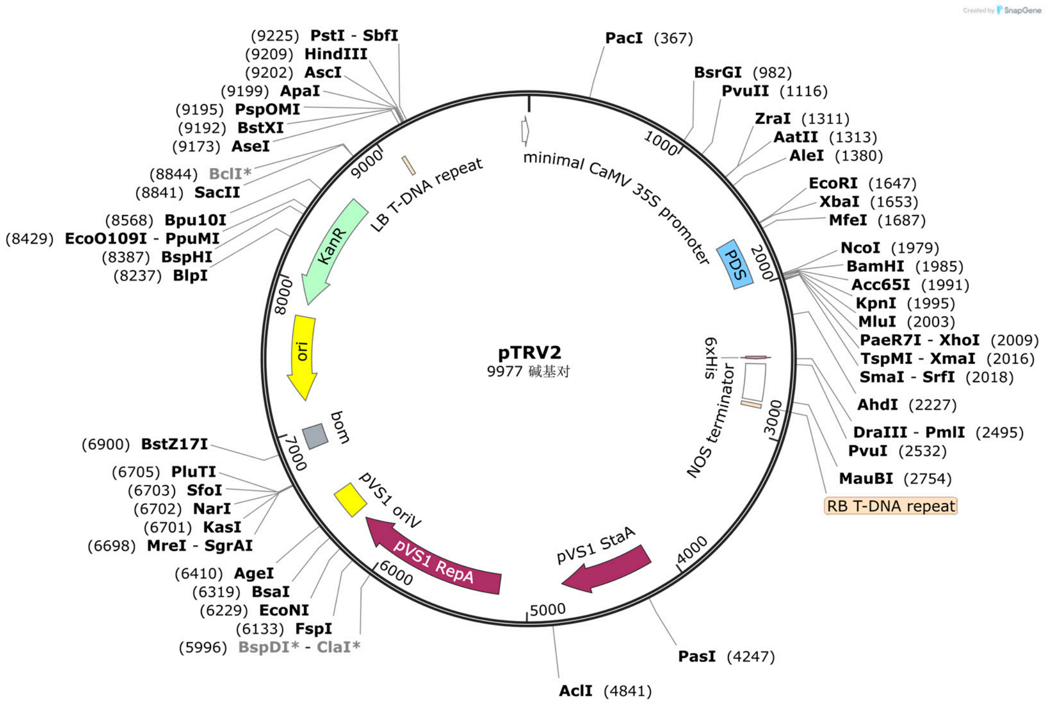

Supplementary Figure S2 (A). Schematic map of the recombinant pTRV2-*SvPDS* vector.

Schematic representation of the recombinant pTRV2-*SvPDS* vector constructed for VIGS-mediated silencing of *SvPDS* in *S. villosa*.

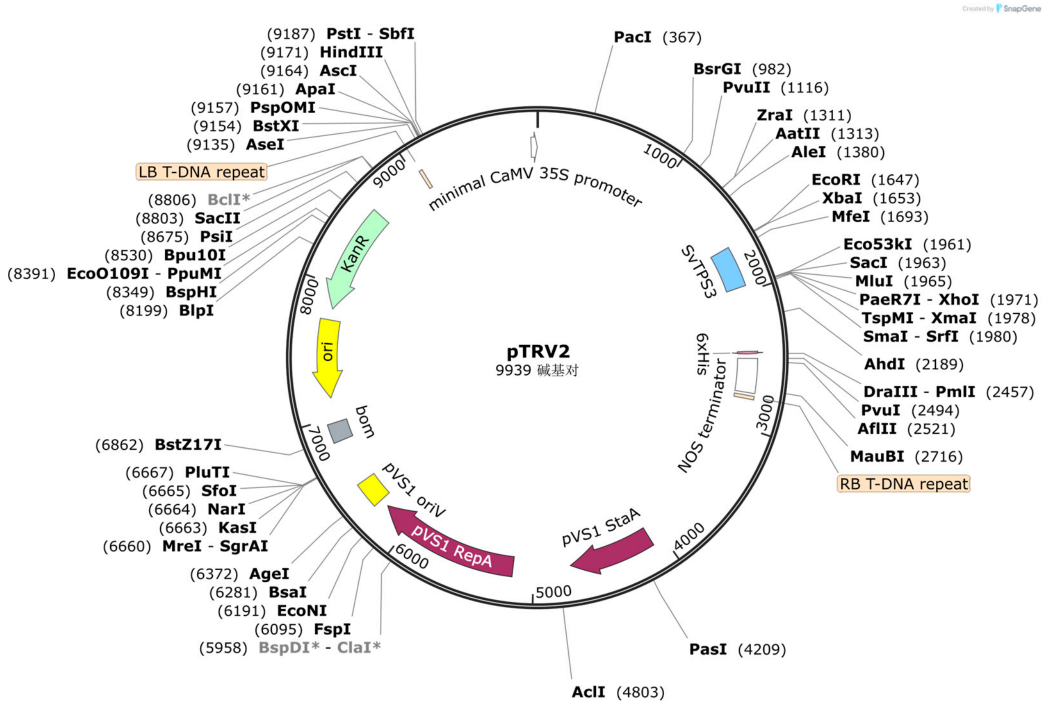

Supplementary Figure S2 (B). Schematic map of the recombinant pTRV2-*SvTPS3* vector.

Schematic representation of the recombinant pTRV2-*SvTPS3* vector constructed for VIGS-mediated silencing of *SvTPS3* in *S. villosa*.
