# Supplementary material for: Establishment of a Virus-Induced Gene Silencing (VIGS) System in Syringa villosa and Functional Characterization of the Floral Scent-Associated Gene SvTPS3
Source: Plants (Basel). 2026 Oct 16;15(20):3179. doi: 10.3390/plants15203179 (PMC13709108; doi:10.3390/plants15203179)
Supplement: Supplementary file 1 [file plants-15-03179-s001.zip › Supplementary File S1.pdf]

Supplementary File S1

SvPDS VIGS target fragment (300 bp)

5’-GGCAATCCACCAGAAAGACTTTGCATGCCAATTGTTGACCATATTACTTCAAAAGGTGGA
GAAATCCTGCTTAATTCACGAATACAAAAGATTGAGCTAAATAAAGACAGCAGTGTTAAG
AACTTTGTGCTAAATGATGGGAGTATGGTTGAAGGAGATGTTTATGTATTTGCAACCCCG
GTTGATATCCTTAAGCTCCTTTTGCCTGATGACTGGAAAGAGATTCAGTATTTCCGAAAA
TTGGATAAATTAGTTGGAGTTCCAGTTATAAATGTTCACATATGGAGCTCACTTCTCAGT
GTGTATGCTGACATGTCTGT-3’

SvTPS3 VIGS target fragment (300 bp)

5’-TATTGAAGCATACAGTAAAAAAGATGAGGCAGGTCAATTGCTACTTAAACTAGCGAAATT
GGATTTCAACATGGTCCAGTCCATGCTTCAAAGAGATCTCCAACAAGTCTCATGTTGGTG
GAAGGATGTGGGCTTGGCAAATAAGTTGTACTTTGCAAGAGATAGGCTGATGGAATGCTT
CTTTTGGTCAGTTGGAATGGTATTTGAACCACAATTTAGTGAATGCCGAAAAGGTTTAAC
CAAAGTGGCTAAGTTAATCACTATAATAGACGATGTTTATGATGTCTATGGCTCTCTTGA-3’
